# A High-Density SNP Map of Sunflower Derived from RAD-Sequencing Facilitating Fine-Mapping of the Rust Resistance Gene *R_12_*

**DOI:** 10.1371/journal.pone.0098628

**Published:** 2014-07-11

**Authors:** Zahirul I. Talukder, Li Gong, Brent S. Hulke, Venkatramana Pegadaraju, Qijian Song, Quentin Schultz, Lili Qi

**Affiliations:** 1 Department of Plant Sciences, North Dakota State University, Fargo, North Dakota, United States of America; 2 Department of Plant Pathology, North Dakota State University, Fargo, North Dakota, United States of America; 3 Northern Crop Science Laboratory, USDA- Agricultural Research Service, Fargo, North Dakota, United States of America; 4 BioDiagnostics Inc., River Falls, Wisconsin, United States of America; 5 Soybean Genomics and Improvement Lab, USDA- Agricultural Research Service, Beltsville, Maryland, United States of America; Nanjing Forestry University, China

## Abstract

A high-resolution genetic map of sunflower was constructed by integrating SNP data from three F_2_ mapping populations (HA 89/RHA 464, B-line/RHA 464, and CR 29/RHA 468). The consensus map spanned a total length of 1443.84 cM, and consisted of 5,019 SNP markers derived from RAD tag sequencing and 118 publicly available SSR markers distributed in 17 linkage groups, corresponding to the haploid chromosome number of sunflower. The maximum interval between markers in the consensus map is 12.37 cM and the average distance is 0.28 cM between adjacent markers. Despite a few short-distance inversions in marker order, the consensus map showed high levels of collinearity among individual maps with an average Spearman's rank correlation coefficient of 0.972 across the genome. The order of the SSR markers on the consensus map was also in agreement with the order of the individual map and with previously published sunflower maps. Three individual and one consensus maps revealed the uneven distribution of markers across the genome. Additionally, we performed fine mapping and marker validation of the rust resistance gene *R_12_*, providing closely linked SNP markers for marker-assisted selection of this gene in sunflower breeding programs. This high resolution consensus map will serve as a valuable tool to the sunflower community for studying marker-trait association of important agronomic traits, marker assisted breeding, map-based gene cloning, and comparative mapping.

## Introduction

Sunflower (*Helianthus annuus* L) is a member of the Asteraceae family, and is the fourth most economically important annual crop grown worldwide for edible oil [1]. Cultivated sunflower is a diploid species (2n = 2x = 34) with a large genome size of ∼3.5 Gb [2]. Molecular markers and high density genetic linkage maps are important tools for understanding genome organization, and can facilitate comparative genomics, marker-assisted selection (MAS), identification of marker-trait associations via linkage or association mapping analysis, and isolation of genes by map-based cloning [3], [4]. Existing marker resources in sunflower include random amplified polymorphic DNA (RAPD) [5], restriction fragment length polymorphism (RFLP) [6]–[8], amplified fragment length polymorphism (AFLP) [9], and simple sequence repeat (SSR) [10]–[12]. Many important agronomic traits, including vertical disease resistance genes [13]–[21], fertility restoration genes [22]–[25] and numerous quantitative trait loci (QTL) were mapped using these data [26]–[31]. While the reports of sunflower linkage maps are numerous (http://sunflower.uga.edu/cmap/), the limited number of markers makes it difficult to conduct fine-scale linkage mapping and map-based cloning. Association mapping and genomic selection are dependent on a large number of polymorphic markers. These analyses are only successful if thousands of markers are available, because of the low level of linkage disequilibrium (LD) present in germplasm resources of sunflower [32]–[35]. When large numbers of markers are employed in an analysis, especially for routine breeding purposes such as genomic selection, the marker must also be high-throughput and cost effective to provide timely and repeatable data.

Single nucleotide polymorphisms (SNP) are the most common type of genetic variation [36]. Through advances in sequencing technologies and high-throughput genotyping facilities, SNP markers have gained much interest in the scientific and breeding community because of their efficiency, repeatability, and low cost [37]. SNPs are usually biallelic and characterized by low mutation rates; therefore, stable from generation to generation across the genome [38]. This stability coupled with the abundance of SNPs makes them very useful both for linkage and genetic diversity studies. SNPs make it possible to conduct genome wide association mapping in low LD species. While SNP studies have been common for some time in human genetics, the advances in sequencing technology have allowed large scale SNP discovery also in crop plant species, such as sunflower [39]–[40]. Recently a high density linkage map based on ∼10,000 SNP markers was reported [41]. The National Sunflower Association (NSA) SNP Consortium, a private-public partnership of commercial seed companies, U.S. Department of Agriculture-Agricultural Research Service (USDA-ARS), and the National Sunflower Association, has developed 10,000 SNP markers using restriction site associated DNA (RAD) protocols and Illumina/Solexa paired-end sequencing chemistry [40]. The development of these SNP markers benefits the sunflower research community as a molecular genetics and genomics resource that offers the promise of speedy, inexpensive genotyping for multiple purposes, but in particular will facilitate gene mapping studies.

Construction of a consensus map from multiple linkage maps offers the opportunity to map a larger number of markers than would be possible in any individual bi-parental map and also tends to eliminate many large marker gaps. Statistical software has been developed to pool segregation data from individual populations and compute loci orders and genetic map distances based on mean recombination frequencies and combined LOD scores [42]. In the absence of the whole genome sequence and a physical map, the high resolution genetic map remains an essential resource for dissection of complex traits and an essential guide to genomics-assisted crop improvement [43]. Consensus maps have been developed using multi-population linkage maps in several crop species including sunflower [41], tomato [43], soybean [44], common bean [45], sorghum [46], red clover [47], and rye [48]. Here, we report the construction of three linkage maps using SNP markers developed from RAD tag sequences and SSR markers previously positioned in the sunflower SSR reference map [10], [11], and the development of a consensus map. We also report and validate a marker linked to a rust resistance gene, *R_12_*, in the constructed consensus map.

## Materials and Methods

### Plant materials

Three mapping populations were used to develop SNP genetic maps in sunflower (Table 1). Five parental lines were chosen to construct these three mapping populations, all but one of which were used in initial RAD tag sequencing [40]. Crosses were made in pairs predicted to maximize total cumulative polymorphism. The first mapping population (Pop1) consisted of 139 F_2_ progeny derived from a cross between HA 89 and RHA 464. HA 89 (PI 599773) is an oilseed maintainer line and RHA 464 (PI 655015) is an oilseed restorer sunflower germplasm which is known to possess resistance genes for both downy mildew and rust diseases [49]. This population was previously used to map the rust resistance gene (*R*-gene) *R*_12_ to linkage group (LG) 11 of sunflower using simple sequence repeat (SSR) markers [13]. The second mapping population (Pop2) consisted of 141 F_2_ progeny derived from a cross between a proprietary confection B line (Nuseed Americas, Woodland, CA, USA) and RHA 464. The third mapping population (Pop3) consisted of 142 F_2_ progeny derived from a cross between CR 29 (Nuseed Americas, Woodland, CA, USA) and RHA 468 (PI 667184). CR 29 is a proprietary confection restorer line and RHA 468 is an oilseed restorer line. To graphically explain relationships among the parent lines, Jaccard's genetic similarity coefficient [50] was calculated using SNP marker data and a dendogram was constructed using unweighted pair-group method of arithmetic averages (UPGMA) clustering analysis in NTSYS-pc version 2.2 [51].

**Table 1:** SNP and SSR marker distributions in the three component maps and the consensus map of sunflower.

	Pop1 (HA 89×RHA 464) F_2_					Pop2 (B-line×RHA 464) F_2_			Pop3 (CR29×RHA 468) F_2_			Consensus map						
Linkage groups	No. of markers			Map length cM	Density cM/marker	No. of SNP markers	Map length cM	Density cM/marker	No. of SNP markers	Map length cM	Density cM/marker	No. of markers			Map length cM	Density cM/marker	No. of large gaps	
	SSR	SNP	Total									SNP	SSR	Total			5 to 10 cM	>10 cM
LG1	9	285	294	88.56	0.30	337	73.82	0.22	33	54.97	1.67	384	9	393	76.09	0.19	3	0
LG2	9	82	91	80.22	0.88	172	73.92	0.43	34	40.59	1.19	214	9	223	81.99	0.37	2	1
LG3	6	122	128	105.89	0.83	185	88.99	0.48	145	88.72	0.61	327	6	333	95.33	0.29	0	0
LG4	6	142	148	57.45	0.39	94	100.30	1.07	156	108.94	0.70	273	6	279	102.45	0.37	4	0
LG5	10	179	189	100.96	0.53	241	91.77	0.38	146	82.09	0.56	374	10	384	91.87	0.24	0	0
LG6	2	51	53	48.03	0.91	117	58.80	0.50	67	56.58	0.84	168	2	170	62.99	0.37	0	0
LG7	8	62	70	67.28	0.96	72	66.09	0.92	60	55.18	0.92	140	8	148	68.31	0.46	3	0
LG8	8	214	222	67.66	0.30	166	62.97	0.38	172	81.40	0.47	320	8	328	75.42	0.23	1	0
LG9	10	108	118	106.79	0.91	179	86.50	0.48	228	108.83	0.48	352	10	362	104.60	0.29	3	0
LG10	13	386	399	94.84	0.24	437	94.48	0.22	95	76.61	0.81	503	13	516	90.89	0.18	1	1
LG11	10	142	152	76.32	0.50	103	88.37	0.86	117	95.86	0.82	246	10	256	99.82	0.39	1	0
LG12	8	141	149	19.84	0.13	142	62.96	0.44	98	66.12	0.67	255	8	263	67.00	0.25	0	0
LG13	4	44	48	31.19	0.65	248	69.45	0.28	136	77.70	0.57	296	4	300	72.93	0.24	1	0
LG14	1	43	44	38.56	0.88	160	78.56	0.49	191	73.63	0.39	285	1	286	76.47	0.27	3	0
LG15	5	54	59	53.95	0.91	158	80.28	0.51	95	84.78	0.89	225	5	230	85.46	0.37	2	0
LG16	5	77	82	105.80	1.29	223	95.77	0.43	144	107.67	0.75	333	5	338	101.28	0.30	1	0
LG17	4	36	40	21.37	0.53	202	97.94	0.48	206	57.52	0.28	324	4	328	90.94	0.28	3	0
Total	118	2168	2286	1164.71	0.51	3236	1370.97	0.42	2123	1317.19	0.62	5019	118	5137	1443.84	0.28	28	2

A total of 548 sunflower lines were used in the present study to validate the *R_12_* specific markers. They include 238 inbred lines and 63 germplasm lines released by USDA, and 247 plant introduction (PI) lines originally collected from 32 countries, which together represent a diverse germplasm pool of cultivated sunflower (Tables S1 and S2).

### DNA extraction and genotyping

Genomic DNA of Pop1 along with its parental lines, HA 89 and RHA 464, were obtained from a previous mapping project [13]. Genomic DNA of Pop2 and Pop3, along with their parents and 548 sunflower germplasm lines, were extracted using 40 mg of lyophilized young leaves with the DNeasy 96 Plant Kit (Qiagen, Valencia, CA, USA) and a modified protocol. Tissue was pulverized with 3-mm steel beads in a Harbil 5G-HD paint shaker (Fluid Management, IL, USA). Buffer AP1 with DX and RNaseA was added to the tissue, 500 µl per sample, and incubated at 55°C for 60 min. Buffer AP2 was added at 150 µl per well, and incubated at −20°C for 15 min. AP3/E was combined with supernatant, 600 µl and 400 µl respectively, and then added to the binding plates. The rest of the extraction was carried out according to kit instructions. DNA was eluted in a final volume of 50 µl, and was quantified using the PicoGreen kit (Molecular Probes) according to the kit instructions. A standard curve was made using quantified λ DNA from 100 to 0 ng/µl. A 1/200 dilution of Picogreen reagent in 1×TE (provided in kit) was mixed with 2 µl of isolated DNA, briefly vortexed, and incubated in the dark for 5 min. Assays were performed in black 96-well Fluotrac plates and fluorescence was measured with a Spectramax Gemini XPS (Molecular Devices) at 485 nm excitation and 538 nm emission wavelengths.

SSR markers were used only in Pop1 in order to determine the linkage groups and map orientation corresponding to the published maps of Tang et al. [10] and Yu et al. [11]. A total of 870 published SSR markers [10]–[12] were screened for polymorphism between the two parents, HA 89 and RHA 464 [13]. Two-hundred fifteen polymorphic SSR markers covering 17 linkage groups were selected for genotyping the 139 F_2_ individuals of Pop1.

A total of 8,723 SNP markers selected from the original 10,000 SNPs derived from RAD sequencing were used to genotype all the parents and F_2_ progenies of the three mapping populations, as well as 548 sunflower lines (Tables S1 and S2). The SNP sequences for the 10,000 targeted loci were presented in Table S3. SNP marker discovery using paired-end RAD sequencing and Illumina Infinium quality control parameters have been described by Pegadaraju et al. [40], SNP markers were named starting with NSA followed by a six digit number. Samples were genotyped with a custom assembled Illumina Infinium chip (Illumina Inc., San Diego, CA, USA) containing 8,723 SNP markers. The genotypic data were analyzed using the Genome Studio Genotyping Module v1.0 (Illumina Inc.) clustering algorithm. All data were visually inspected and manually rescored if any errors were evident in the calling of the homozygous or heterozygous clusters. To reduce data set complexity in line with software requirements, SNP data were filtered to remove uninformative markers, such as those with no polymorphism observed between parents, those where one/both of the parental genotypes failed to amplify in the assay, or those possessing a heterozygous genotype in at least one of the parental genotypes. The remaining SNPs were mapped using JoinMap 4.1 [42].

### Construction of individual population linkage maps

Linkage maps were constructed independently for each mapping population using the same procedures and parameters in each case. All the SNP markers and the majority of the SSR markers used for linkage mapping were co-dominant. The Chi-square test (*p*>0.05) was used to assess goodness-of-fit to the expected segregation ratio for each marker using the ‘locus genotype frequencies’ feature of JoinMap 4.1. Markers that showed significant segregation distortion from the expected 1∶2∶1 (co-dominant) or 3∶1 (dominant) ratios were excluded from map construction. Markers were assigned to linkage groups applying the independence LOD (logarithm of the odds) parameter with LOD threshold values ranging from 2.0 to 12.0. We used the ‘similarity of loci’ command of JoinMap to identify perfectly identical markers (similarity value  = 1.000) which are supposed to be mapped at exactly the same position on the linkage group. In order to reduce the burden of calculation effort, only one marker was kept of the ‘similar loci’ for linkage mapping analysis. Linkage analysis and marker order were carried out using the regression mapping algorithm. Recombination fractions were converted to map distances in centimorgans (cM) using the Kosambi mapping function [52]. The excluded similar markers, however, were included in the final map. The linkage groups of individual maps were drawn using MapChart 2.2 [53]. All unique, two-way, and three-way sets of shared markers across three mapping populations were analyzed and visualized using the Venn diagram [54].

### Construction of a consensus linkage map

The integration of the linkage groups derived from three mapping populations followed the principle described by Stam [55] using JoinMap 4.1 [42]. First, groupings and group nodes for each individual population were loaded into the navigation tree of the same JoinMap 4.1 project. The groups that correspond to the same linkage group with at least two common loci were combined into a single ‘combined group node’ in the navigation tree using the ‘Combine groups for map integration’ command. The integrated linkage map was constructed using a regression mapping algorithm with the same threshold parameters used for individual population linkage mapping. The graphical representation of the integrated linkage map was drawn using MapChart 2.2 [53].

### Comparison of the consensus map with the individual linkage maps

The extent of collinearity in marker orders between consensus and component genetic maps was assessed by calculating the Spearman's rank correlation coefficients (ρ) from marker positions in consensus and individual genetic maps. Significance tests were conducted in R version 2.13.1 [56]. Comparative analyses of marker order and collinearity were illustrated by plotting marker positions on the consensus map against individual population maps.

### Analysis of marker distribution

All linkage groups were divided into 1, 2, 5, and 10 cM intervals and the observed marker frequency distribution of each interval was calculated. The observed marker frequencies per centiMorgan (cM) unit interval were compared to that of expected frequencies generated from a Poisson distribution using a Chi-square test [57]. The probability density function of the expectation is
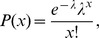
where *x* is the actual marker count in each interval, λ is the average number of markers per interval and *e* is the base of the natural logarithm. Analyses were conducted with the R statistical package [56].

### Assessment of the rust resistance gene *R_12_* and linked SNP markers

Rust phenotypic data of Pop1 were obtained from Gong et al. [13], where Pop1 was first used to map the rust resistance gene *R_12_* with SSR markers. Briefly, urediniospores of North America (NA) race 336 were used to inoculate F_2_ plants, along with the two parental lines HA 89 (susceptible parent) and RHA 464 (resistant parent). Twenty seedlings of each of the F_2_-derived F_3_ families were also phenotyped with the same pathogen race to distinguish between F_2_ plants that were homozygous or heterozygous for the resistance gene. Rust infection types and severity (pustule coverage) were scored 10–12 days after inoculation as described by Qi et al. [16]. The rust phenotypic and SNP marker data were combined for fine-mapping of the gene *R_12_*.

## Results

### Genetic diversity of the parental lines

**Figure 1:**
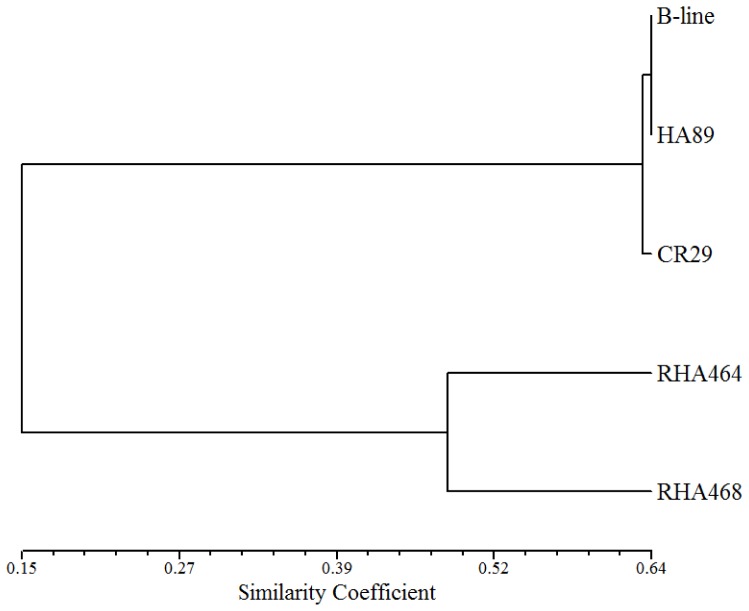
Genetic relationship of mapping parents. Dendogram of the 5 sunflower parents of the three mapping populations based on unweighted pair-group method with arithmetic averages clustering analysis (UPGMA).

Three segregating populations derived from five parental lines were used in this study (Table 1). RHA 464 was a common parent between Pop1 and Pop2. The SNP marker data of the parental lines were used to assess the genetic diversity between the parental lines based on Jaccard's coefficient and a dendogram was constructed using UPGMA clustering analysis (Figure 1). The parental lines of the respective mapping populations varied widely in genetic relatedness, with the parents of Pop2 being the most diverse pair (similarity coefficient value is only 0.007).

### Component maps of individual populations

The three mapping populations, Pop1, Pop2, and Pop3, were used to produce three separate high-density genetic maps containing 2,286, 3,236, and 2,123 markers in each map, respectively (Table 1).

#### Pop1 linkage map

The construction of the Pop1 linkage map started with 141 F_2_ individuals from a cross between HA 89 and RHA 464. Two individuals were discarded because of too much missing data, leaving a total of 139 F_2_ individuals for the final linkage analysis. Pop1 was first genotyped with a total of 220 polymorphic SSR markers. Of these, 118 informative and co-dominant SSRs were integrated with SNP markers to construct a high-density linkage map of Pop1. The number of SSR markers for each LG varied and ranged from one on LG14 to 13 on LG10 (Table 1). All but two SSRs detected a single locus. CRT136 was mapped to LGs 4 and 7, and ORS679 was mapped to LGs 12 and 15, consistent with previous data [10]–[11].

Additionally, a total of 2,413 polymorphic SNP markers were used for linkage mapping in Pop1. About 10.2% of the SNP markers (245/2,413) showed significant distortion (*P*<0.05) from the expected Mendelian ratio, and were discarded from mapping analysis, leaving a total of 2,286 segregating markers (118 SSR and 2,168 SNP) (Table 1). The markers were assembled into 17 LGs identified in the same manner as the genetic maps of Tang et al. [10] and Yu et al. [11]. Mapped SNP markers were distributed in all 17 LGs, although, like the SSRs, the distribution was not homogeneous in all regions. The genetic map covers a total length of 1,164.71 cM, with an average density of one marker in every 0.51 cM. The length of the linkage groups ranges from 19.84 cM in LG12 to 106.79 cM in LG9, and the number of markers per linkage group varies from 40 in LG17 to 399 in LG10 (Table 1). Ninety one percent of the gaps between two adjacent loci were smaller than 5 cM with the largest being 33.89 cM on LG9 (Table S4).

#### Pop2 linkage map

The Pop2 linkage map was constructed with 141 F_2_ individuals of a cross between a proprietary confection B-line and RHA 464. Filtration of the SNP genotype data yielded 3,464 good quality SNP marker data for linkage analysis in Pop2. A total of 228 markers (6.6%) showed significant (*P*<0.05) distortion from the expected 1∶2∶1, which were removed, yielding a final genetic map of 3,236 SNP markers assembled into 17 LGs. The 17 LGs were identified on the basis of the common SNP markers located on each chromosome relative to the linkage map of Pop1. The Pop2 linkage map covers a total length of 1,370.97 cM with an average density of one marker in every 0.42 cM (Table 1). The length of individual linkage groups varies from 58.80 cM in LG6 to 100.30 cM in LG4. The number of markers per linkage group also varies considerably from 72 in LG7 to 437 in LG10 (Table 1). Most of the gaps (94%) between two adjacent loci were smaller than 5 cM while the largest gap was only 23.10 cM on LG4 (Table S4).

#### Pop3 linkage map

Linkage mapping of Pop3 started with 142 F_2_ individuals from a cross between CR 29 and RHA 468, and 2,681 good quality SNP marker data were obtained after filtration of the genotype data. Segregation analysis revealed that 552 SNPs (20.6%) showed significant (*P*<0.05) distortion from the expected Mendelian ratio and were removed from the linkage analysis. The remaining SNP markers were placed onto 17 sunflower linkage groups except for 6 markers, which could not be suitably added in any linkage group, resulting in a final map consisting of 2,123 SNP markers (Table 1). The total length of the genetic map of Pop3 was 1,317.19 cM with an average density of one marker in every 0.62 cM, the lowest among all three maps. Individual linkage groups range from 40.59 cM in LG2 to 108.94 cM in LG4, and the number of markers per linkage group varies from 33 in LG1 to 228 in LG9 (Table 1). Gaps 5 cM or smaller between two adjacent loci accounted for about 93% of the total gaps observed, with the largest gap being 26.87 cM at the distal end of LG13 (Table S4).

#### Unique and common markers across component maps

A total of 608, 1,300, and 855 SNP markers were mapped exclusively in Pop1, Pop2, and Pop3, respectively (Table 2; Figure S1). However, there were 252 SNP markers that were common and mapped in all three mapping populations. In total, 988, 696, and 320 common SNP markers were identified between pairs of component maps Pop1–Pop2, Pop2–Pop3 and Pop1–Pop3, respectively. The large number of common markers found between the Pop1 and Pop2 maps was expected due to RHA 464 being a common parent. Common SNP markers mapped in all three component maps were distributed in all linkage groups ranging from 2 in LG15 to 52 in LG8 (Table 2).

**Table 2:** The unique and common SNP markers across linkage groups and genetic maps of sunflower.

Linkage group	Unique SNP markers in			Common SNP markers between			Common SNP markers across all populations	Total SNPs
	Pop1	Pop2	Pop3	Pop1 and Pop2	Pop2 and Pop3	Pop1 and Pop3		
LG01	35	76	12	240	11	0	10	384
LG02	27	103	14	50	15	1	4	214
LG03	43	105	65	34	35	34	11	327
LG04	69	29	64	19	38	46	8	273
LG05	52	113	37	63	45	44	20	374
LG06	17	65	28	19	24	6	9	168
LG07	27	38	30	15	10	11	9	140
LG08	61	46	33	41	27	60	52	320
LG09	24	73	108	27	63	41	16	352
LG10	50	77	11	281	29	5	50	503
LG11	74	38	42	17	24	27	24	246
LG12	52	30	55	75	29	6	8	255
LG13	10	124	35	26	93	3	5	296
LG14	13	70	100	11	72	12	7	285
LG15	14	80	51	36	40	2	2	225
LG16	27	136	69	26	51	14	10	333
LG17	13	97	101	8	90	8	7	324
Total	608	1,300	855	988	696	320	252	5,019

### Consensus map

**Figure 2:**
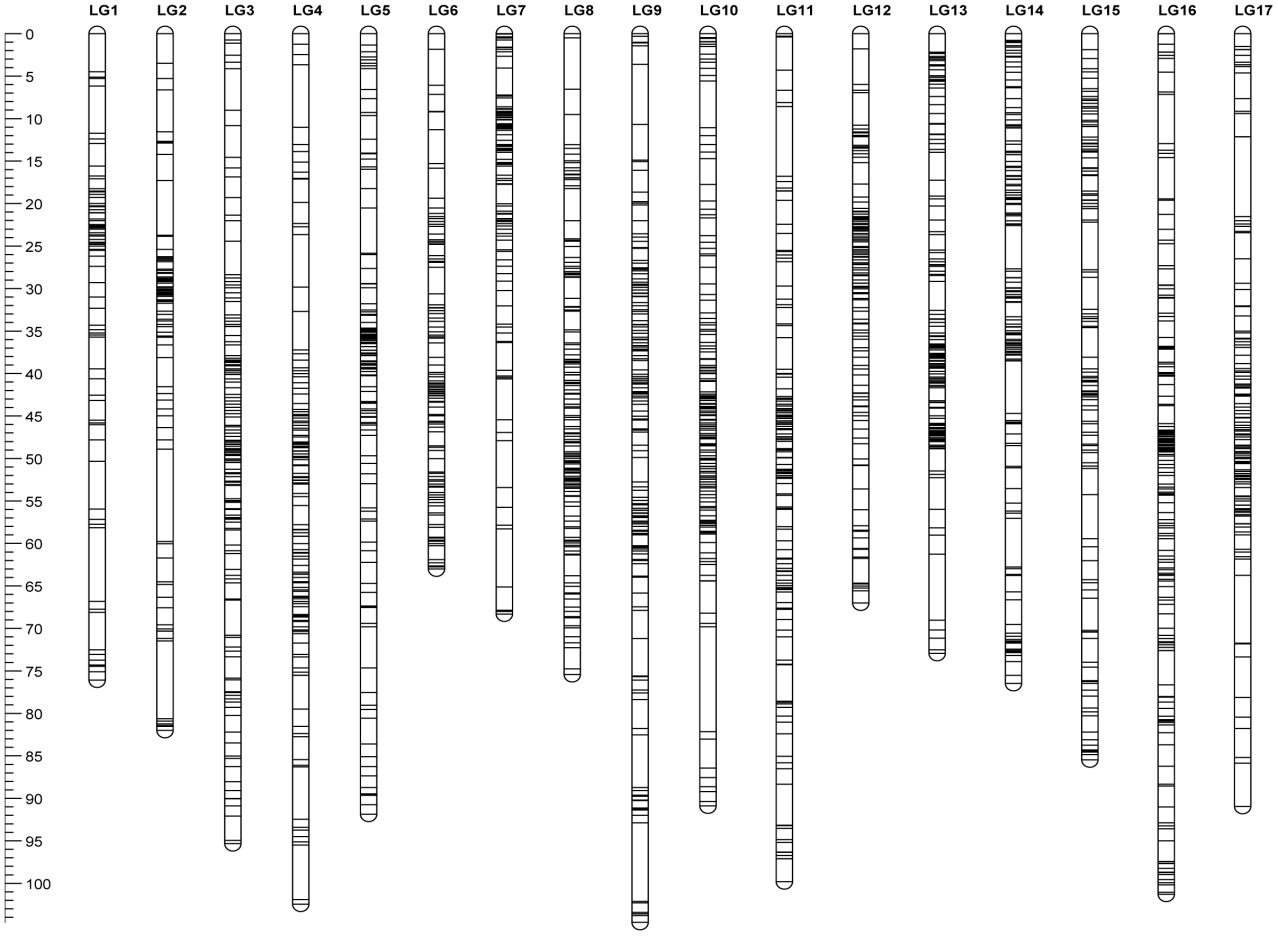
Schematic representation of the consensus map. Ruler on left indicates the cM distance and the horizontal lines across the chromosomes indicate locus positions on each chromosome.

Consensus maps were constructed by merging corresponding linkage groups from the three individual maps, one linkage group at a time, using JoinMap 4.1. The common markers on homologous linkage groups of individual maps served as bridges to integrate maps into a single consensus map. A schematic illustration of the consensus map, which included the expected 17 linkage groups of sunflower, is presented in Figure 2. The integrated linkage map consisted of 5,019 SNP markers and 118 SSR markers with a total map length of 1,443.84 cM (Table 1). The length of the linkage groups ranges from 62.99 cM in LG6 to 104.60 cM in LG9, and the number of markers per linkage group varies from 148 in LG7 to 516 in LG10. The total map length of the consensus map is greater than the map length of each component map. Detailed information of the consensus map including the genetic distance, marker types, and unique and common markers among the populations is illustrated in Figure S2, S3, S4, S5, S6, S7, and Table S4.

#### Collinearity of markers between consensus and component maps

**Figure 3:**
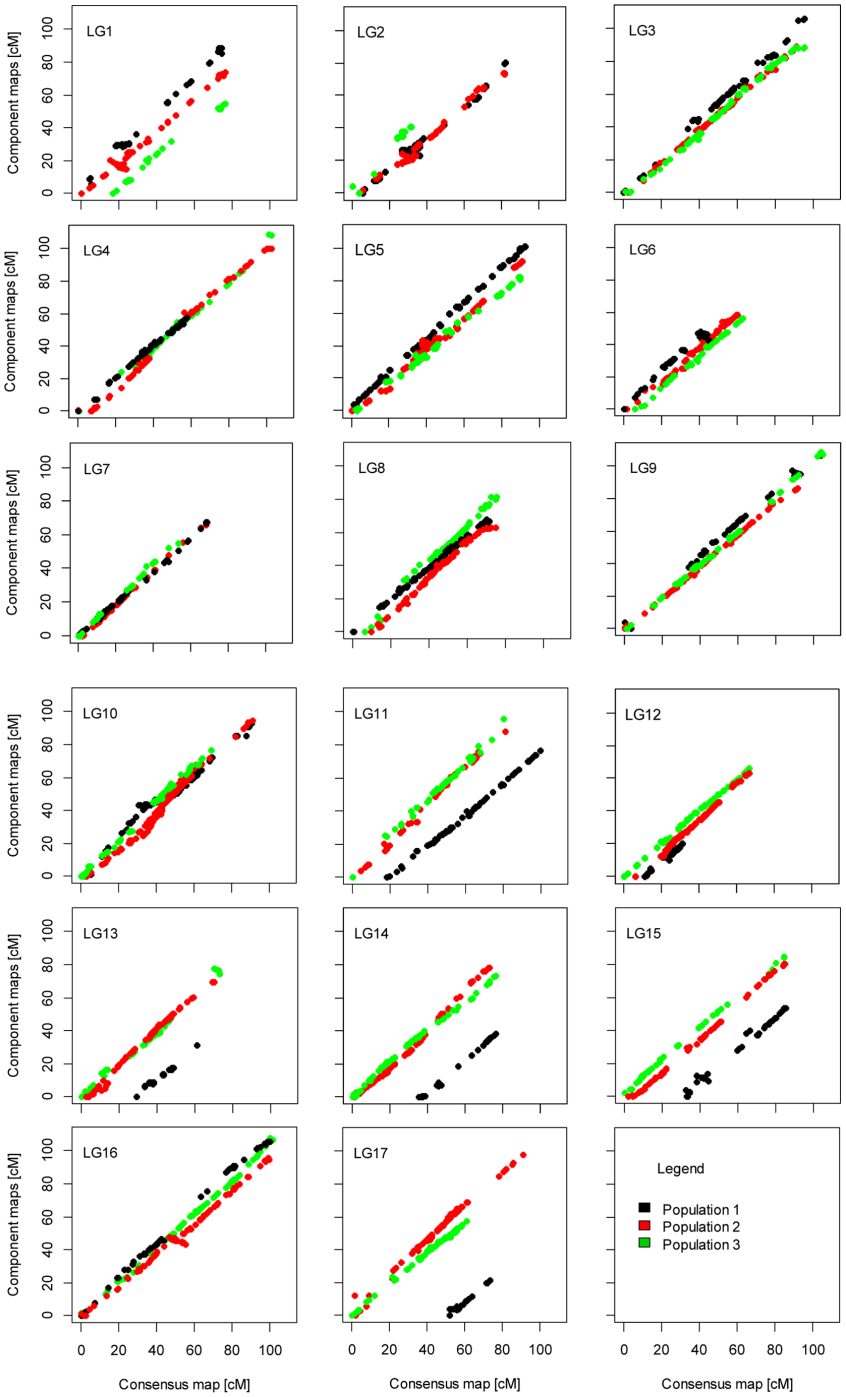
Scatter plots of the 17 sunflower linkage groups showing the collinearity of marker order among the consensus genetic map and component population maps.

Inequality of the lengths of individual linkage groups between the component maps and the consensus map was clearly visible in collinearity plots (Figure 3). In general, marker order between the consensus map and the component maps was consistent across all the linkage groups, with only a few ambiguities identified in LGs 1, 2, 5, 15, and 16. Correlation analysis revealed that marker orders were strongly correlated in all 17 linkage groups between the consensus and component maps, with a mean correlation coefficient value of 0.972 (Table S5).

#### Distribution of markers along linkage groups

**Figure 4:**
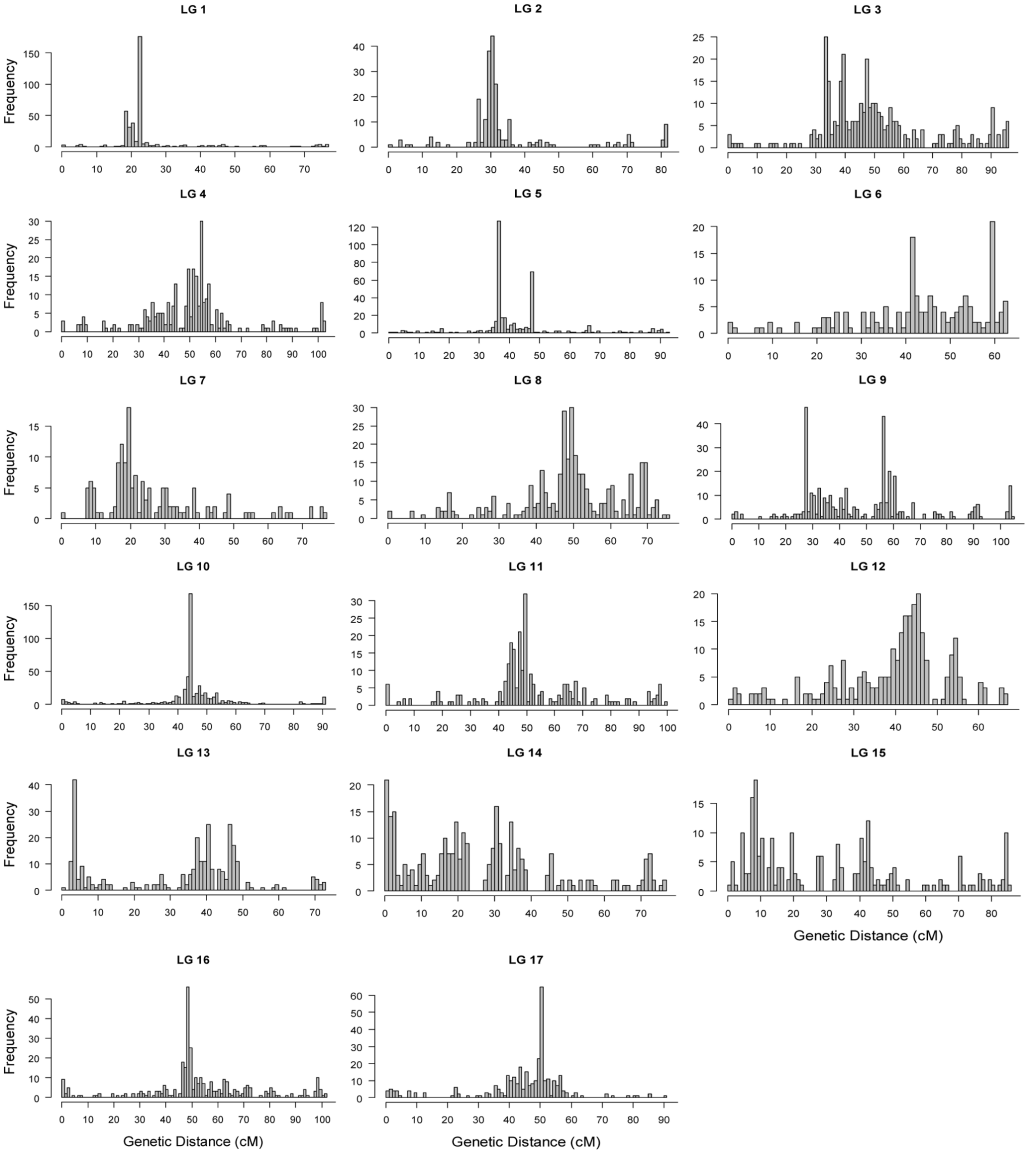
Frequency distribution of markers on the sunflower consensus map. A x-axis indicates genetic distance in each linkage group in 1-cM intervals and the y-axis indicates number of markers in each 1-cM bin.

Chi-square test of marker distribution at 1, 2, 5, and 10 cM intervals on linkage groups revealed highly significant deviations from the Poisson expectation (data not shown). Genome wide marker distribution in 1-cM intervals shows a clear clustering of markers in certain genomic regions, indicating that the markers were not randomly distributed along the entire length of the sunflower linkage groups (Figure 4). The average genetic distance between markers was 0.28 cM and ranged from 0.18 cM in LG10 to 0.46 cM in LG7 (Table 1). Large gaps (>5 cM) observed in most linkage groups of the component maps were reduced during the map integration process. In the consensus map, gaps between two adjacent loci became smaller, with 98.6% (2,141 of 2,171) of the gaps being less than 5 cM (Table 1, Figure 4). There were only two gaps >10 cM, one each on LG2 and LG10 with the largest being 12.37 cM on LG10.

### Fine mapping of the rust resistance gene *R_12_* and marker validation

The rust resistance gene *R_12_*, present in the inbred line RHA 464, was previously mapped to LG11 with flanking SSR markers, CRT275 and ZVG53, in an interval of 10.6 cM [13]. The same F_2_ population (Pop1 in the present study) was used to saturate the *R_12_* region with SNP markers. Rust phenotypic data of Pop1 were integrated with SNP marker data, and seven linked SNP markers were identified, five on one side (NSA_000064, NSA_003320, NSA_003426, NSA_004155, NSA_008884), and two on the other side (NSA_001570, and NSA_001392), defining an interval less than 2.3 cM surrounding the previously mapped *R_12_* gene in LG11 (Table S4).

Five of seven linked SNP markers were used to genotype each of the 548 lines in our validation set (Tables S1 and S2). Among 348 sunflower lines with SNP data, the RHA 464-specific SNP allele of NSA_004155 is only present in RHA 464 and did not exist in any other of the tested lines (Table 3). Another SNP, NSA_003426, which had genotypic data in 322 lines was homozygous for a unique allele in RHA 464 and was heterozygous for that same allele in PI 600809. However, two other SNP markers, NSA_000064 and NSA_008884, which co-segregated with NSA_003426 and NSA_004155, shared the RHA 464 alleles with more than 250 rust susceptible lines, and were not diagnostic markers for *R_12_*. NSA_001570 was located on the other side of *R_12_* and 23 susceptible lines shared the RHA 464 allele (Table 3). The diagnostic alleles for *R_12_* at NSA_003426 and NSA_004155 are cytosine nucleotides while the alleles in HA 89 are adenine nucleotides. Comparison of SNP alleles and rust phenotypes in a subset of 32 lines which included four rust resistant lines carrying different *R*-genes and 28 susceptible lines confirmed the cytosine alleles of NSA_003426 and NSA_004155 were diagnostic for *R_12_* (Table 4). These results indicated that the rust resistance gene *R_12_* is probably not widely distributed in the sunflower germplasm pool and the two SNPs could serve as diagnostic markers for the gene *R_12_* in most genetic backgrounds.

**Table 3:** Validation of SNP markers linked to the rust gene *R_12_* in a diverse sunflower germplasm pool.

SNP	Map location (cM)	Summary				
		No. of lines genotyped	No. of lines with data	No. of HA 89 allele	No. of RHA 464 allele	No. of heterozygous allele
NSA_000064	44.55	548	527	209	287	31
NSA_008884	44.55	548	332	37	266	29
NSA_003426	44.55	548	322	320	1	1
NSA_004155	44.55	548	348	347	1	0
*R_12_*	45.38	-	-	-	-	-
NSA_001570	46.78	548	315	288	23	4

**Table 4:** A subset of rust phenotypes and SNP profiles of NSA_003426 and NSA_004155.

Lines	Rust phenotypes**	Rust R-genes	SNP genotypes		References
			NSA_003426	NSA_004155	
HA 89*	S		AA	AA	[13], [58]
RHA 464*	R	*R_12_*	CC	CC	[13], [58]
HA-R3	R	*R_4_*	AA	AA	[16], [58]
HA-R6	R	*R_13a_*	AA	AA	[14], [58]
HA-R8	R	unknown	AA	AA	[13], [58]
Rf ANN-1742	R	*R_11_*	AA	AA	[18], [58]
HA 61	S		AA	AA	[58]
HA 285	S		AA	AA	[58]
HA 286	S		AA	AA	[58]
HA 304	S		AA	AA	[58]
HA 305	S		AA	AA	[58]
HA 315	S		AA	AA	[58]
HA 317	S		AA	AA	[58]
HA 318	S		AA	AA	[58]
HA 323	S		AA	AA	[58]
HA 460	S		AA	AA	[58]
HA-R1	S		AA	AA	[58]
HA-R5	S		AA	AA	[58]
HA-R7	S		AA	AA	[58]
RHA 265	S		AA	AA	[58]
RHA 270	S		AA	AA	[58]
RHA 271	S		AA	AA	[58]
RHA 272	S		AA	AA	[58]
RHA 273	S		AA	AA	[58]
RHA 279	S	*R_1_*	AA	AA	[21], [58]
RHA 282	S		AA	AA	[58]
RHA 298	S		AA	AA	[58]
RHA 340	S	*R_adv_*	AA	AA	[19], [21], [58]
RHA 801	S		AA	AA	[58]
RHA 854	S		AA	AA	[58]
RHA 855	S		AA	AA	[58]
RHA 856	S		AA	AA	[58]
RHA 858	S		AA	AA	[58]
RHA 859	S		AA	AA	[58]

*HA 89 and RHA 464 were susceptible and resistant parents of Pop1 used in the present study.

**rust phenotypic data were taken from Qi et al. (58) using NA rust races 336 and 777.

## Discussion

Construction of a linkage map is often the first step to characterizing the genome of an organism. We presented a high density integrated genetic linkage map of sunflower using ∼8,700 SNP markers derived from RAD-sequencing. Three F_2_ mapping populations were developed using five parental lines of cultivated sunflower, four of which were used in the initial RAD sequencing step. Genetic analysis revealed that a high degree of genetic diversity exists between the parents of all three mapping populations. This contributed to the high SNP density in each of the component maps. These maps can be more readily used for breeding purposes because they contain SNPs that are informative within the closely related gene pool of cultivated sunflower. In addition, the high density genetic map facilitated fine mapping of the rust resistance gene *R_12_*, providing closely linked SNP markers for high throughput, marker-assisted selection of this gene in breeding programs.

The individual F_2_ mapping populations in our study are almost identical in size with ∼140 individuals per population. The linkage maps of Pop2 and Pop3 were similar in length (1,371 and 1,317 cM, respectively), while the Pop1 map (1,165 cM in length) was somewhat shorter than the rest of the maps. Comparisons of linkage groups among individual maps revealed that the upper ends of LGs 4 (∼42 cM), 13 (∼29 cM), 14 (∼33 cM), 15 (∼28 cM) and 17 (∼50 cM) and the lower end of LG12 (∼36 cM) in the Pop1 map showed no marker coverage, though the same regions in the other two maps possessed many mapped loci (Table S4). The other larger gap that lacked mappable markers was∼34 cM in LG9 of Pop1 as well as a few other ∼20 cM gaps in various LGs of all three mapping populations. Bowers et al. [41] also reported several gaps of up to 26 cM in individual sunflower crosses. This pattern is not due to a lack of SNP markers on these chromosome segments but is likely due to the mapping parents sharing similar genomic regions identical by descent. Large gaps with low polymorphism were also observed in the linkage maps of other species like soybean [44], common bean [45], sorghum [46] and rye [48].

Segregation distortion is a ubiquitous phenomenon in crop species that skews the frequency of alleles from the expected Mendelian ratio within a segregating population and has strong impact in genetic map construction [9], [59]–[63]. In the present study, the proportion of distorted markers varied from 6.6% (Pop2) to 20.6% (Pop3) which is comparable to other species like sorghum [46], red clover [47], rye [48], maize [64], and pigeonpea [65]. The distribution of distorted markers among individual linkage groups in the component maps was distinctly different. In Pop1, the highest number of distorted markers was observed in LGs 1, 7, 10, and 12, whereas in Pop2, the marker distorted regions were present in LGs 4, 9, 11, and 12. In Pop3, where the highest percentage of skewed segregations was observed, the most distorted markers were in LGs 1, 2, 8, 9, and 10. In order to smooth integration of the consensus map, we discarded 254, 228 and 552 distorted markers, respectively, during individual map construction. However, 48.2%, 67.7% and 75.7% of these distorted markers from Pop1, Pop2, and Pop3, respectively, were eventually included in the consensus map through integration of information available from one or both of the other populations without segregation distortion. Our mapping strategy with multiple segregating populations thus offered an excellent basis for the development of a consensus map in sunflower.

The presence of common markers among component maps is a prerequisite for building a consensus linkage map [66]. In this study, sufficient numbers of common SNP markers were segregating within each individual mapping population, which made the merging of a large number of markers into the final consensus map possible. High numbers of common markers, with stable recombination frequencies across component mapping populations, allow positioning of markers on a highly reliable reference map and also in regions that were poorly covered in the individual maps [67]. As a result, the final consensus map possesses 5,137 markers (118 SSR and 5,019 SNP) spanning 1,443.84 cM, divided amongst 17 linkage groups (the actual number of sunflower haploid chromosomes), with an average distance of 0.28 cM between adjacent markers. The map length is comparable to the combined sunflower map (1,310 cM) developed recently by Bowers et al. [41] with 10,080 marker loci. The total length of the consensus map was greater than the length of each of the individual maps (Table 1). The extended map length of the consensus map was mainly due to the addition of markers to the distal parts of some linkage groups. Additionally, the consensus map allowed us to fill most of the larger gaps on individual maps, reducing the number of gaps >10 cM from 10 to 18 on the individual maps to just two on the consensus map.

Despite minor local inversions of neighboring markers in linkage groups 1, 2, 15, and 16, the collinearity of the marker order between the consensus map and the individual maps showed excellent congruence (Figure 3). The high value of Spearman's rank correlation of marker orders between the consensus and individual maps supports this finding (Table S5). Local inversion of closely spaced markers is a common feature during map integration [46]–[47], [68]–[71]. Short span marker order rearrangements could be the reflection of real genetic events or could be caused by statistical uncertainty due to many weak linkages [68], a small number of progeny studied [72], or heterogeneity of recombination frequencies among populations. In the present study, marginal shifts in marker order were found in highly dense marker regions. Resolving scrambled marker order at high-density regions of the genome would require extremely large mapping populations [68]. Alternatively, the issue of inversions would be resolved by comparing linkage maps with a physical map of the sunflower genome which is yet to be completed [73].

The distribution of markers along linkage groups was not random and there were marker-rich and marker-poor regions in the sunflower linkage maps (Figures 2 and 4). Highly dense marker regions are typically centromeric regions [74]–[75]. However, this is not conclusive in our study, as many of the linkage groups showed more than one region of high marker density (Figure 4), similar to the finding of Bowers et al. [41]. A total of 118 publicly available SSR markers are present in our consensus map, which allowed us to reference the homologous linkage groups to the published sunflower maps. Overall, the order of the SSR markers was well conserved in all linkage groups between our map and the published sunflower SSR maps [10]–[12] and also the other integrated sunflower SNP map [41].This would allow cross-referencing between different published sunflower maps and would offer the opportunity of exploring a much larger number of markers for a given genomic region.

A total of three SNP markers, two common between Pop1 and Pop3 and one between Pop1 and Pop2, map to different linkage groups in alternative populations, which could only be explained by the existence of paralogous loci. Similar paralogous loci were observed in sorghum [46] and most recently in sunflower [41] when aligning genetic linkage maps derived from four mapping populations. The number of paralogous loci reported by Bowers et al. [41] was much higher (∼14%) than we observed in our study. The most likely explanation for this difference might lay in the SNP development strategies between the two studies. In the linkage mapping of Bowers et al. [41], the SNPs were designed based on deep EST sequencing of the parental lines. The paralogous loci are the result of gene duplication, and the ESTs of paralogs would have similar sequences in some cases, causing non-specific binding of the SNP primers. In the current study, SNPs were developed from sunflower genomic sequence using restriction site-associated DNA sequencing (RAD-Seq), which is based on identifying polymorphic variants adjacent to restriction enzyme recognition sites [40].

The use of SNP markers combined with publicly available SSR markers in multiple populations greatly increased marker saturation in our consensus map, a major improvement over the low resolution sunflower maps constructed with single populations and other marker types [5]–[12]. The present consensus map of 5,019 SNP and 118 SSR markers is the second most dense genetic linkage map in sunflower next to the one developed recently by Bowers et al. [41] with 10,080 markers. Our consensus map can serve as a valuable tool to sunflower breeders for marker-trait association in QTL or association mapping of important agronomic traits, marker assisted breeding, map-based gene cloning, and comparative mapping.

## Supporting Information

Table S1 **List of three-hundred one USDA released sunflower germplasms used for marker validation.**
(XLSX)

Table S2 **List of two-hundred forty seven sunflower plant introduction (PI) lines used for marker validation.**
(XLSX)

Table S3 **SNP sequences for the 10,000 targeted loci.**
(XLSX)

Table S4 **SNP and SSR marker positions in Pop1, Pop2, Pop3, and consensus genetic linkage maps.**
(XLSX)

Table S5 **Spearman's rank correlation coefficients between marker positions in the consensus map and individual population maps in each linkage group of sunflower.**
(DOCX)

Figure S1 **A three-way Venn diagram illustrating all unique, two-way and three-way sets of shared SNP markers mapped in three component populations.** The mapping populations are abbreviated as in the text: Pop 1 = HA 89×RHA 464; Pop 2 = B-Line×RHA 464; Pop 3 = CR29×RHA 468.
(TIF)

Figure S2 **Integrated genetic linkage map of sunflower.** The map shows the linkage groups 1, 2, and 3 developed from three F_2_ mapping populations. Markers in bold font are SSR markers.
(TIF)

Figure S3 **Integrated genetic linkage map of sunflower.** The map shows the linkage groups 4, 5, and 6 developed from three F_2_ mapping populations. Markers in bold font are SSR markers.
(TIF)

Figure S4 **Integrated genetic linkage map of sunflower.** The map shows the linkage groups 7, 8, and 9 developed from three F_2_ mapping populations. Markers in bold font are SSR markers.
(TIF)

Figure S5 **Integrated genetic linkage map of sunflower.** The map shows the linkage groups 10, 11, and 12 developed from three F_2_ mapping populations. Markers in bold font are SSR markers.
(TIF)

Figure S6 **Integrated genetic linkage map of sunflower.** The map shows the linkage groups 13, 14, and 15 developed from three F_2_ mapping populations. Markers in bold font are SSR markers.
(TIF)

Figure S7 **Integrated genetic linkage map of sunflower.** The map shows the linkage groups 16 and 17 developed from three F_2_ mapping populations. Markers in bold font are SSR markers.
(TIF)
