# Supplementary material for: A High-Density SNP Map of Sunflower Derived from RAD-Sequencing Facilitating Fine-Mapping of the Rust Resistance Gene R12
Source: PLoS One. 2014 Jul 11;9(7):e98628. doi: 10.1371/journal.pone.0098628 (PMC4094432; doi:10.1371/journal.pone.0098628)
Supplement: Table S5 — Spearman's rank correlation coefficients between marker positions in the consensus map and individual population maps in each linkage group of sunflower. (DOCX) [file pone.0098628.s005.docx]

**Table S5** Spearman's rank correlation coefficients between marker positions in the consensus map and individual population maps in each linkage group of sunflower

| **LGs** | **Maps** | **Pop2** | **Pop3** | **Consensus** |
| --- | --- | --- | --- | --- |
|  | Pop1 | 0.792 | 0.8657 | 0.884 |
| **1** | Pop2 |  | 0.972 | 0.896 |
|  | Pop3 |  |  | 0.983 |
|  | Pop1 | 0.742 | 0.816 | 0.836 |
| **2** | Pop2 |  | 0.652 | 0.810 |
|  | Pop3 |  |  | 0.952 |
|  | Pop1 | 0.995 | 0.991 | 0.992 |
| **3** | Pop2 |  | 0.993 | 0.998 |
|  | Pop3 |  |  | 0.996 |
|  | Pop1 | 0.998 | 0.994 | 0.995 |
| **4** | Pop2 |  | 0.998 | 0.996 |
|  | Pop3 |  |  | 0.996 |
|  | Pop1 | 0.993 | 0.945 | 0.993 |
| **5** | Pop2 |  | 0.998 | 0.986 |
|  | Pop3 |  |  | 0.977 |
|  | Pop1 | 0.92 | 0.827 | 0.821 |
| **6** | Pop2 |  | 0.998 | 0.992 |
|  | Pop3 |  |  | 0.999 |
|  | Pop1 | 0.997 | 0.985 | 0.997 |
| **7** | Pop2 |  | 0.993 | 0.998 |
|  | Pop3 |  |  | 0.982 |
|  | Pop1 | 0.999 | 0.999 | 0.999 |
| **8** | Pop2 |  | 0.999 | 0.998 |
|  | Pop3 |  |  | 0.997 |
|  | Pop1 | 0.991 | 0.944 | 0.993 |
| **9** | Pop2 |  | 0.999 | 0.999 |
|  | Pop3 |  |  | 0.999 |
|  | Pop1 | 0.999 | 0.998 | 0.995 |
| **10** | Pop2 |  | 0.999 | 0.997 |
|  | Pop3 |  |  | 0.998 |
|  | Pop1 | 0.99 | 0.998 | 0.999 |
| **11** | Pop2 |  | 0.996 | 0.998 |
|  | Pop3 |  |  | 0.997 |
|  | Pop1 | 0.968 | 0.906 | 0.971 |
| **12** | Pop2 |  | 0.999 | 0.999 |
|  | Pop3 |  |  | 0.995 |
|  | Pop1 | 0.967 | 0.949 | 0.938 |
| **13** | Pop2 |  | 0.997 | 0.999 |
|  | Pop3 |  |  | 0.998 |
|  | Pop1 | 0.982 | 0.986 | 0.982 |
| **14** | Pop2 |  | 0.998 | 0.999 |
|  | Pop3 |  |  | 0.998 |
|  | Pop1 | 0.977 | 0.949 | 0.972 |
| **15** | Pop2 |  | 0.996 | 0.999 |
|  | Pop3 |  |  | 1.000 |
|  | Pop1 | 0.997 | 0.998 | 0.998 |
| **16** | Pop2 |  | 0.906 | 0.919 |
|  | Pop3 |  |  | 1.000 |
|  | Pop1 | 0.783 | 0.718 | 0.772 |
| **17** | Pop2 |  | 0.993 | 0.998 |
|  | Pop3 |  |  | 0.997 |
