# Supplementary figures and images for: A High-Density SNP Map of Sunflower Derived from RAD-Sequencing Facilitating Fine-Mapping of the Rust Resistance Gene R12
Source: PLoS One. 2014 Jul 11;9(7):e98628. doi: 10.1371/journal.pone.0098628 (PMC4094432; doi:10.1371/journal.pone.0098628)

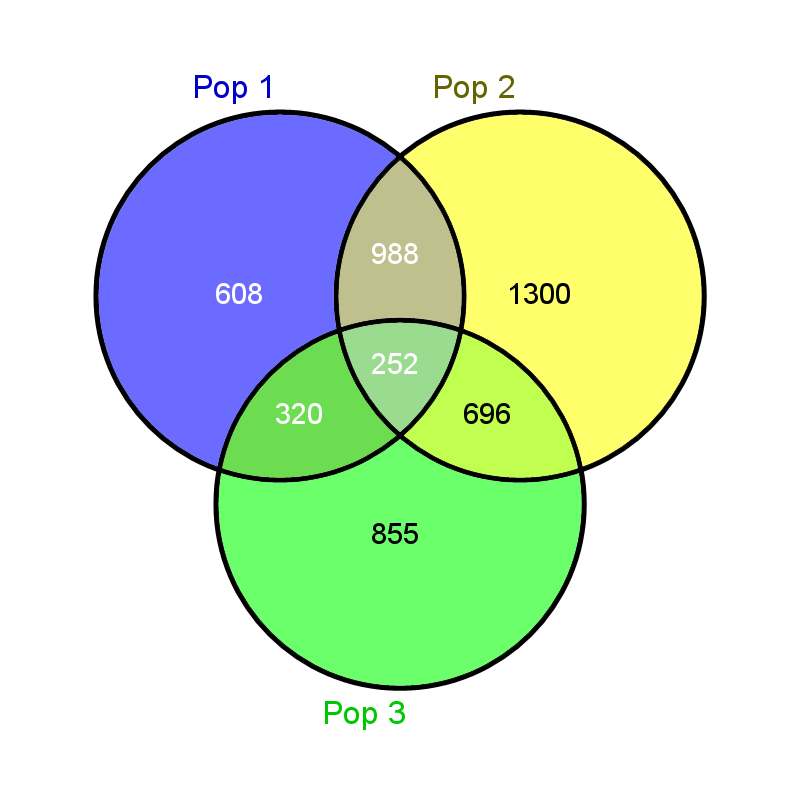

Supplement: Figure S1 — A three-way Venn diagram illustrating all unique, two-way and three-way sets of shared SNP markers mapped in three component populations. The mapping populations are abbreviated as in the text: Pop 1 = HA 89×RHA 464; Pop 2 = B-Line×RHA 464; Pop 3 = CR29×RHA 468. (TIF) [file pone.0098628.s006.tif]

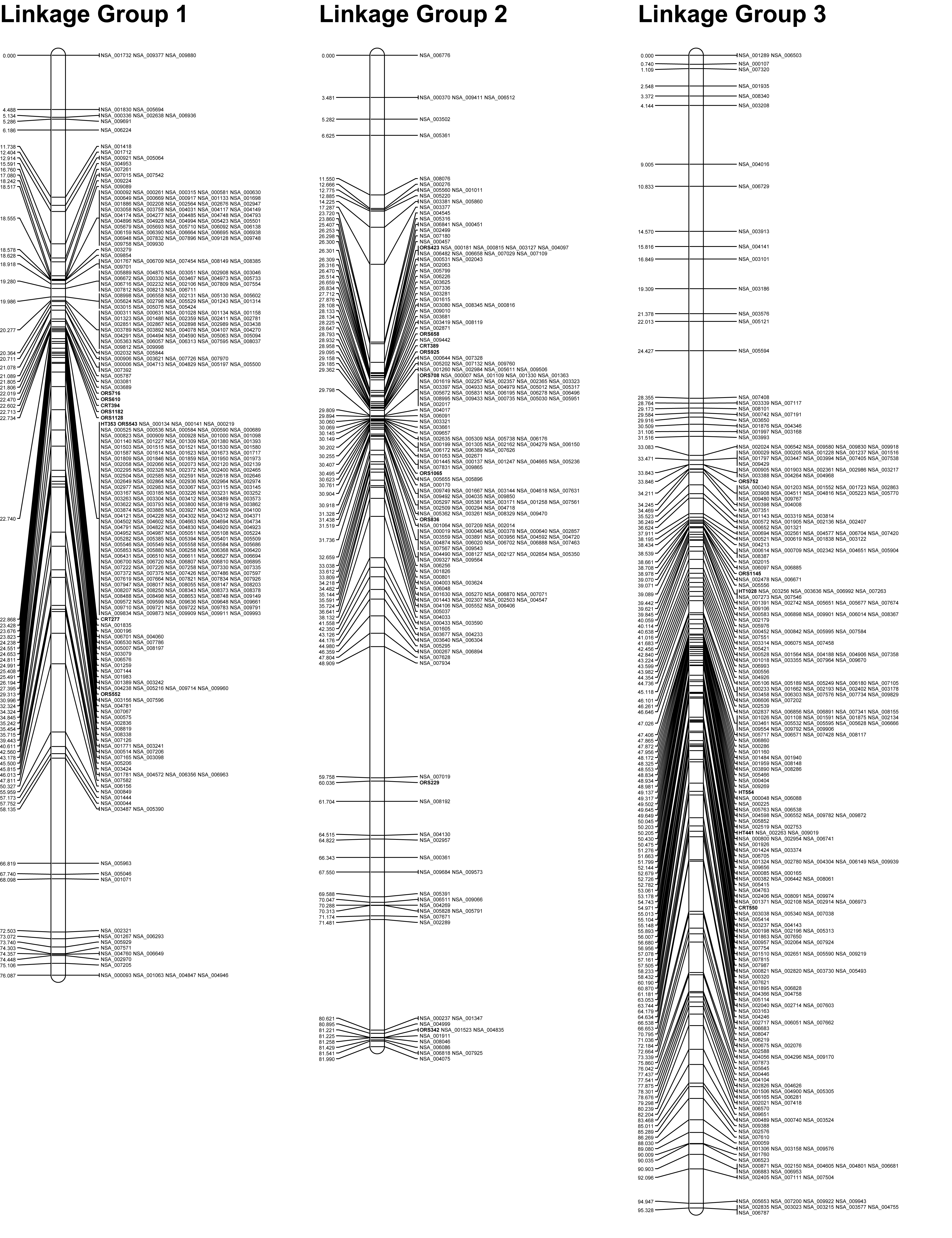

Supplement: Figure S2 — Integrated genetic linkage map of sunflower. The map shows the linkage groups 1, 2, and 3 developed from three F2 mapping populations. Markers in bold font are SSR markers. (TIF) [file pone.0098628.s007.tif]

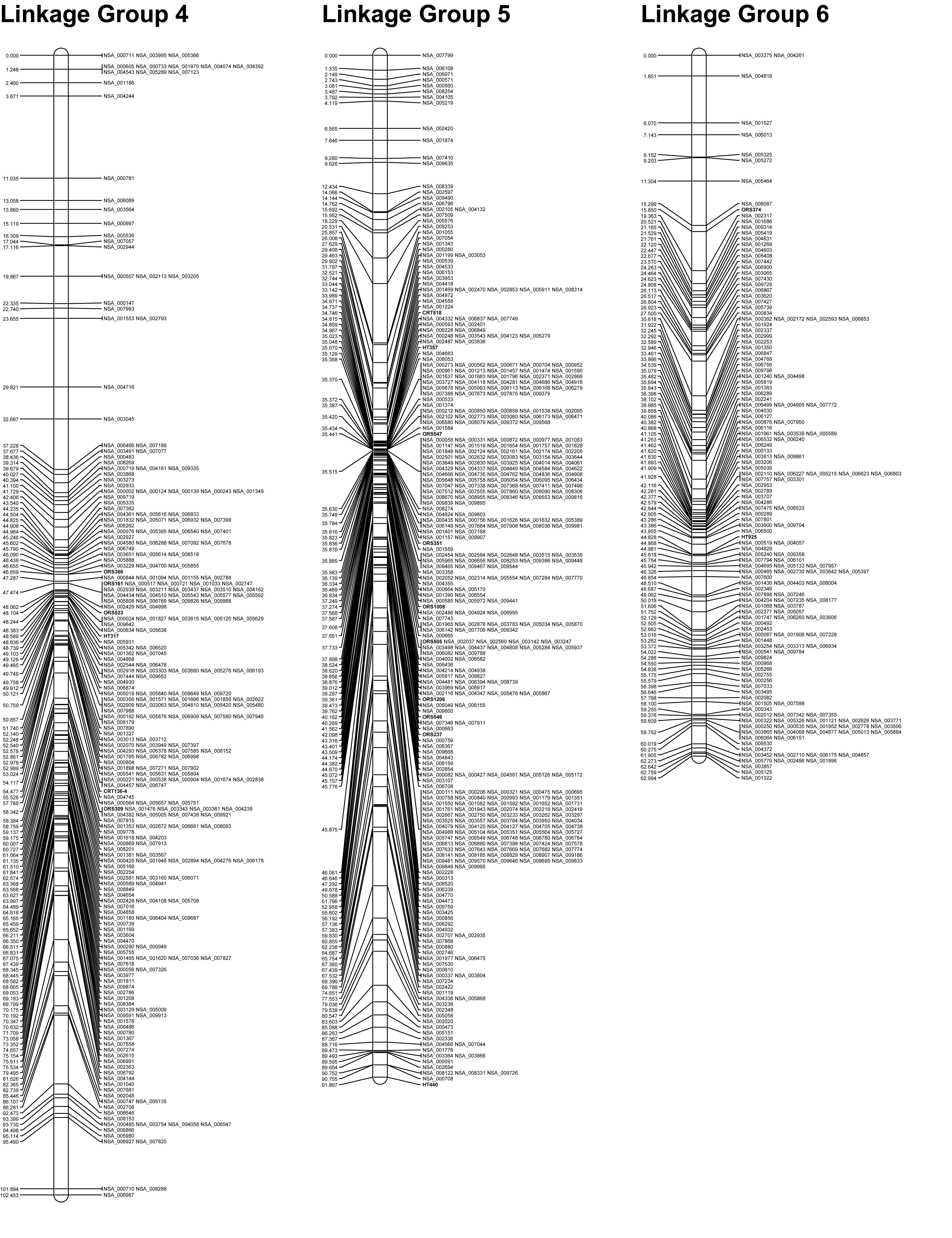

Supplement: Figure S3 — Integrated genetic linkage map of sunflower. The map shows the linkage groups 4, 5, and 6 developed from three F2 mapping populations. Markers in bold font are SSR markers. (TIF) [file pone.0098628.s008.tif]

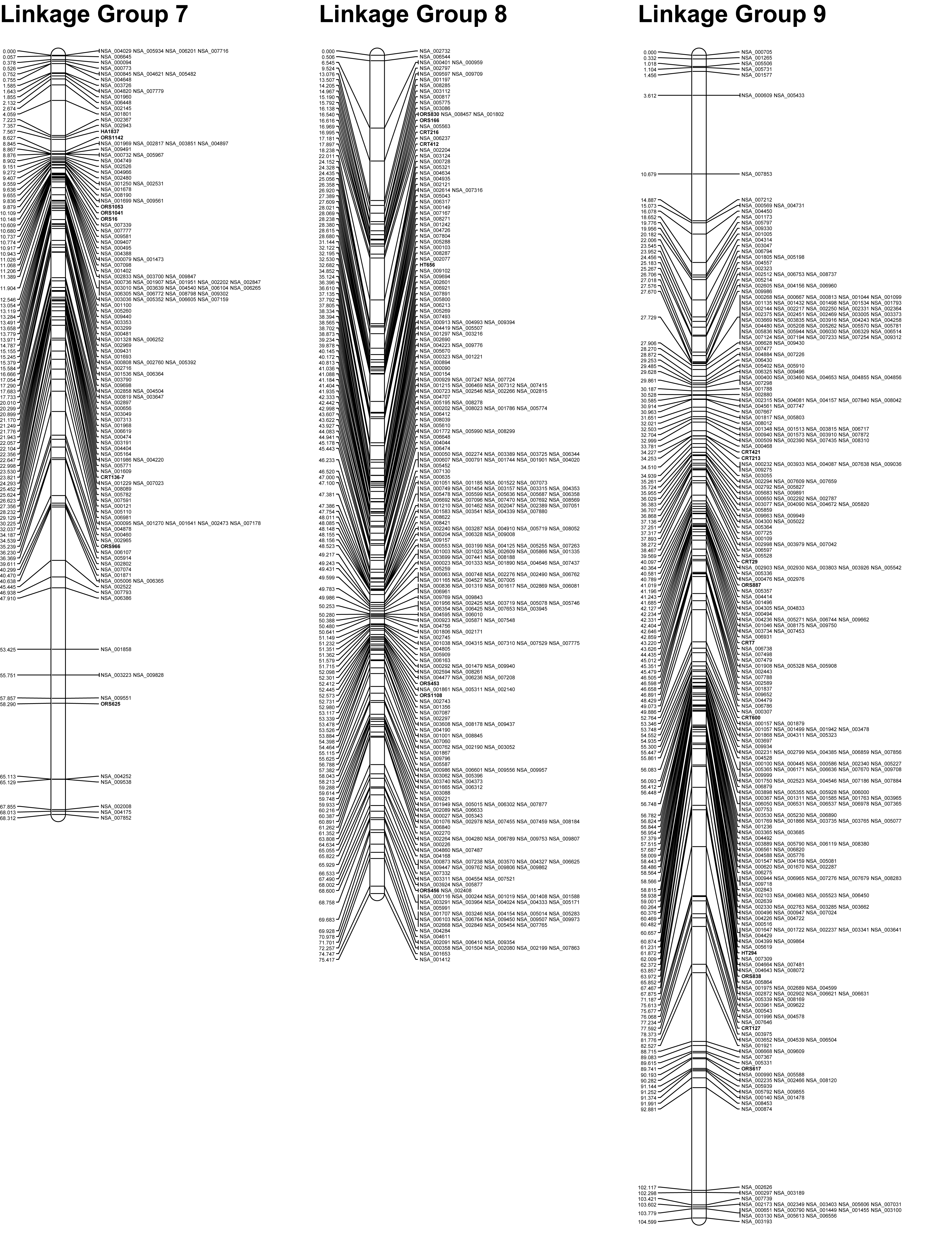

Supplement: Figure S4 — Integrated genetic linkage map of sunflower. The map shows the linkage groups 7, 8, and 9 developed from three F2 mapping populations. Markers in bold font are SSR markers. (TIF) [file pone.0098628.s009.tif]

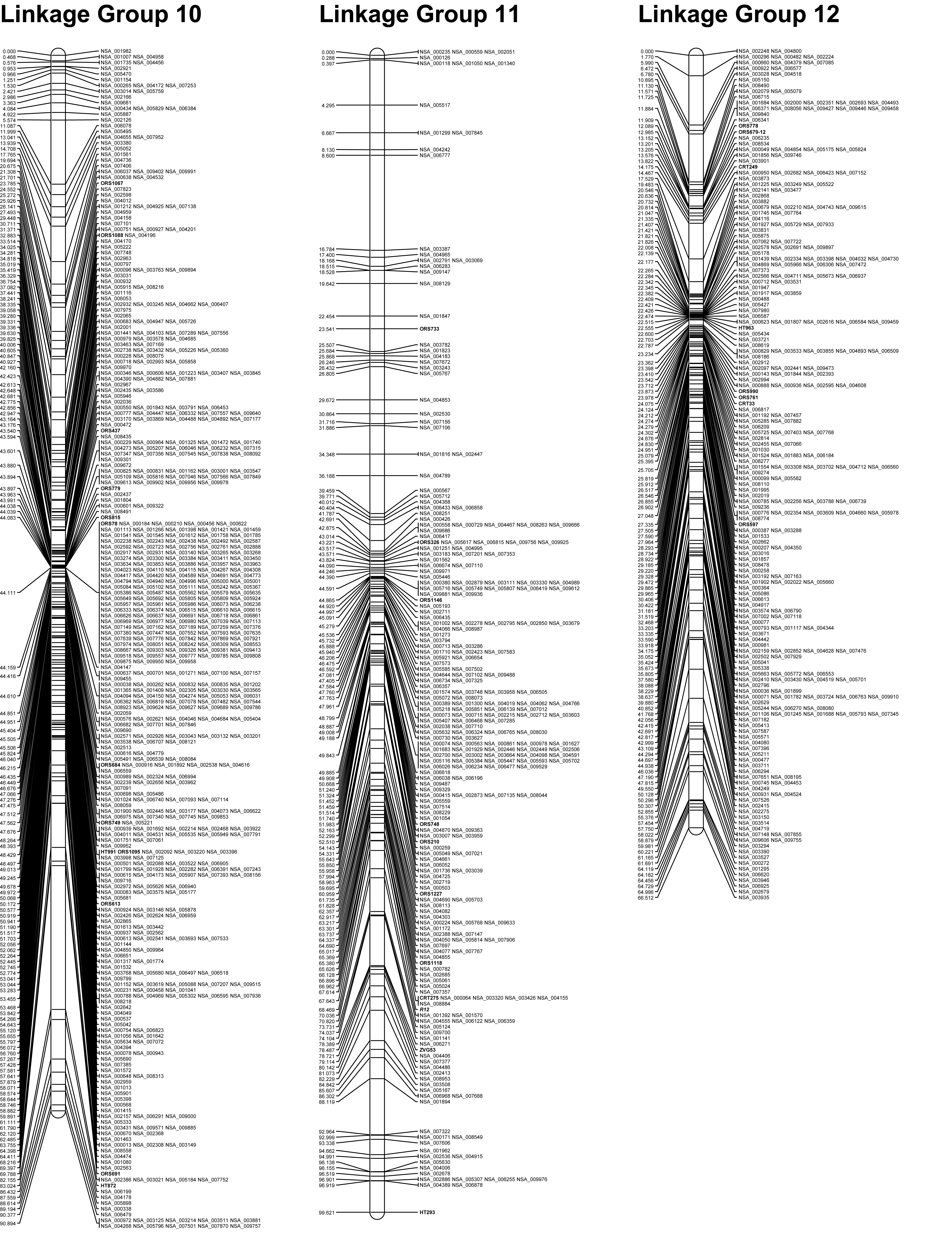

Supplement: Figure S5 — Integrated genetic linkage map of sunflower. The map shows the linkage groups 10, 11, and 12 developed from three F2 mapping populations. Markers in bold font are SSR markers. (TIF) [file pone.0098628.s010.tif]

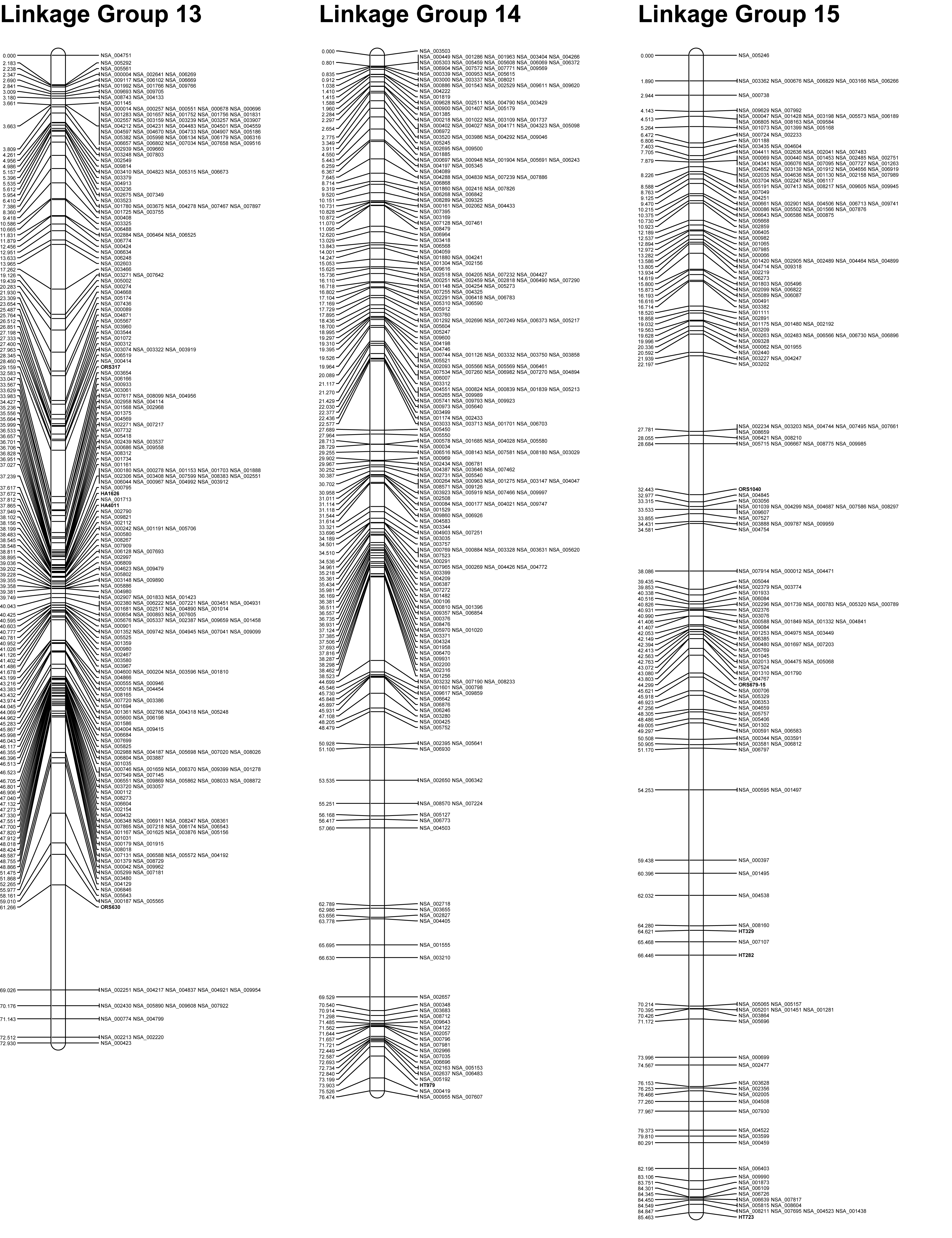

Supplement: Figure S6 — Integrated genetic linkage map of sunflower. The map shows the linkage groups 13, 14, and 15 developed from three F2 mapping populations. Markers in bold font are SSR markers. (TIF) [file pone.0098628.s011.tif]

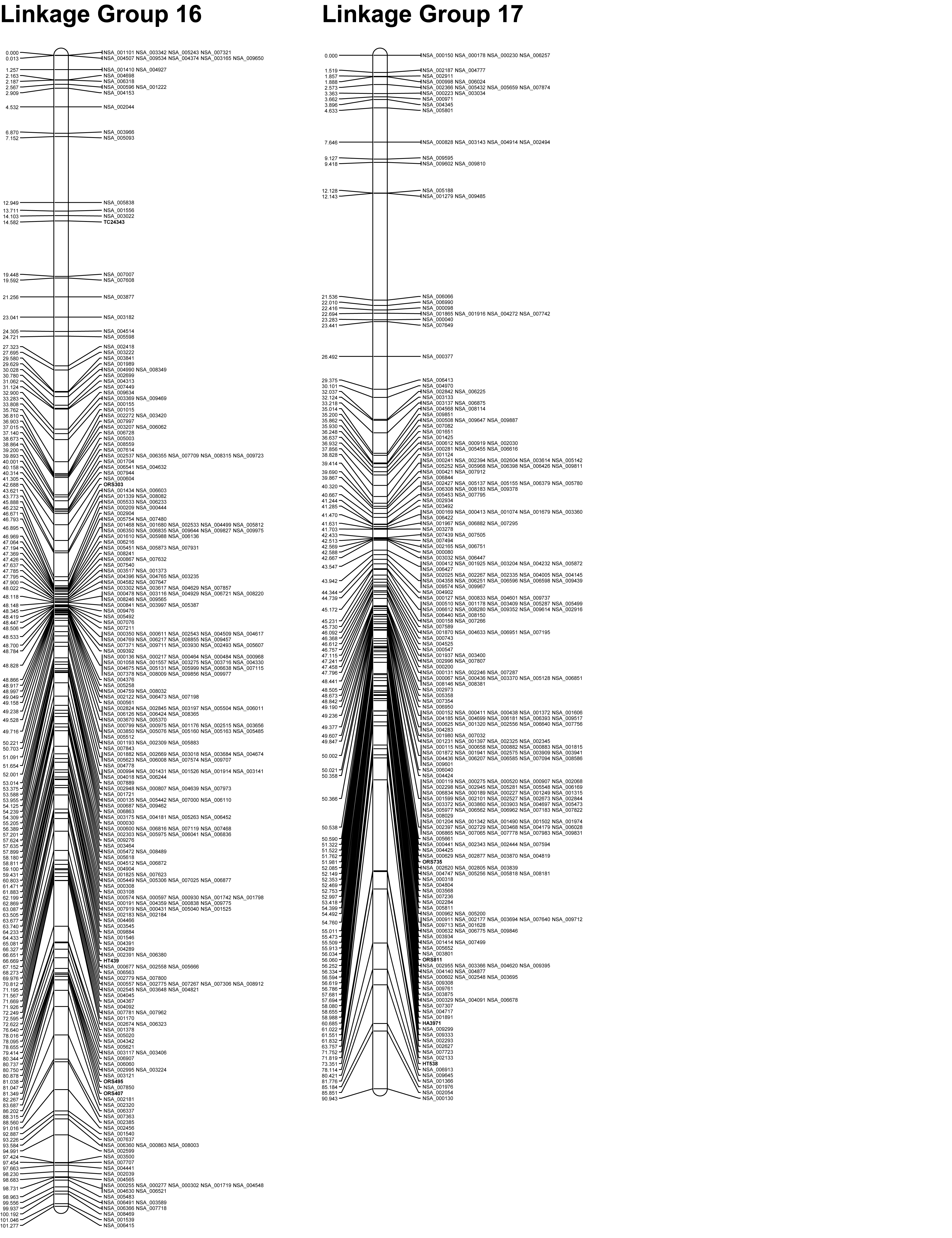

Supplement: Figure S7 — Integrated genetic linkage map of sunflower. The map shows the linkage groups 16 and 17 developed from three F2 mapping populations. Markers in bold font are SSR markers. (TIF) [file pone.0098628.s012.tif]
